# Quantifying left ventricular trabeculae function – application of image-based fractal analysis

**DOI:** 10.1002/phy2.68

**Published:** 2013-09-10

**Authors:** Brandon Moore, Lakshmi Prasad Dasi

**Affiliations:** Department of Mechanical Engineering, Colorado State UniversityFort Collins, Colorado, 80523-1374

**Keywords:** CT imaging, ejection, fractal, trabeculae

## Abstract

The ventricular-blood interface is geometrically complex due to the presence of ventricular trabeculae carneae (VTC). We introduce a new image-based framework to quantify VTC function using high-resolution computed tomography (CT) imaging and offer new insights into the active role of VTCs during ejection. High-resolution Cine CT scans of a patient with normal cardiac function were acquired at a resolution of 0.77 mm per pixel at 10 phases of the cardiac cycle. The images were segmented and the VTC surface was obtained by triangulating the segmented data. Fractal dimension of the VTC surface was calculated for each cardiac phase as a function of scale size using the box-counting algorithm. The fractal dimension, *D* corresponding to VTCs ranged between 2.05 and 2.2 and varied as a function of time during the cardiac cycle. Fractal dimension is highest at diastole and lowest at peak systole with the change being significantly different (*P* < 0.003). This variation of *D* when plotted against stroke volume (i.e., D-V loop) revealed an active VTC role due to hysteresis in the loop. Physically the hysteresis in the D-V loop indicates a new mechanical function of VTCs as structures that provide mechanical leverage during early systolic ejection through contraction. VTC relaxation is noted to occur during late diastole at larger ventricular volume. D-V loop of VTCs quantifies VTC function. A new dynamic physical role of VTCs is suggested by way of mechanical leverage, as opposed to the traditionally accepted passive role.

## Introduction

In this study, we introduce a technique to determine and quantify active ventricular trabeculae carneae (VTC) function through clinical computed tomography (CT) imaging and examine VTC contributions to systolic and diastolic function of the ventricle. While quantifying macroscopic or gross properties of the pumping chambers and heart valves, such as size, overall shape, and valve performance indices are well established spanning over many imaging modalities, no quantitative noninvasive image-based methodology exists to characterize the function of the small-scale fingerlike projections known as trabeculae carneae. Trabeculations are particularly prominent in the ventricles and are known to originate during early embryonic development when woven cardiac fibers are “compacted” into a solid, continuous structure (Weiford et al. 2004; Gandhi 2010). However, in some cases, such as the noncompacted ventricle (Petersen et al. 2005), VTCs are so numerous and prominent that the entire interior of the ventricle is filled with myocardial tissue and appears “spongy” associated with significantly diminished pumping performance. While excessive VTCs are not desirable, lack of VTCs can also negatively impact cardiac performance. This is because of the critical role VTCs play to passively improve systolic ejection performance (Burch et al. 1952). Therefore, a new index to accurately capture VTC function may provide clinical utility to further define ventricular function and more importantly aid in clinical management to better characterize ventricular remodeling.

The objective of this study is to apply advanced geometric analysis tools to (1) determine and quantify any dynamic VTC function via high-resolution computed tomography, and (2) report new mechanistic insight that had not been previously conceptualized with respect to VTC dynamics in a healthy heart. By accomplishing both of these aims, a methodology and baseline measurements are established for assessing trabecular function. Furthermore, reducing such measurements to a simple, quantifiable parameter – fractal dimension – could enable clinicians to much more easily interpret complex imaging data.

To quantify VTC function, we utilize fractal geometric principles; in particular, we adopt the notion of fractal dimension of the ventricular inner surface. In general, any smooth surface is a two-dimensional (2D) object with an area associated as a measure of its size. Similarly, a three-dimensional (3D) object is associated with volume as a measure of its size. Nevertheless, nature consists of surfaces that are extremely convoluted in a way that even though these surfaces exist within a finite volume, their surface area is extremely large. The ventricle contains such a surface, and while it has not before been studied using fractal geometry, it is an excellent candidate for such analysis. This is due to its complex topology, subtle changes in which are virtually undetectable without the use of fractal tools. Examples of other cardiovascular structures or phenomena with similar geometric properties that have been analyzed via fractal geometry include the lung (Buchman 2012; Copley et al. 2012), arterial branching (Bui et al. 2009; Moledina et al. 2011; Nakamura et al. 2011), and myocardial perfusion (Bassingthwaighte et al. 1989). “Fractal” surfaces in this context are smooth at the scale range of <1 mm, but appear rough without well-defined structure in the 10–100 mm scale range. Such surfaces that have drastically different “roughness” at different length scales are referred to as multifractals. The fractal dimension *D*(λ) is a measure that quantifies precisely the dimensionality of such objects as a function of scale size λ. Thus, a surface which appears 2D at the 1-mm scale range may appear to be more 3D (i.e., more space filling) at a larger scale. To be precise, *D*(λ) quantifies the exact dimensionality of the object, which for the case of the human lung, or the ventricular surface is a number between 1 and 3, and not necessarily equal to the integer value of 2. Such analysis tools have also been previously used to characterize electrocardiogram (ECG) data with clinical utility (Tulppo et al. 2005; Mandel et al. 2012). We believe that the methodology presented here and the preliminary new insights will lead to further detailed analysis of VTC function as a predictor of ventricular function either in the context of cardiac remodeling during load-induced congestive heart failure or remodeling in response to ischemic heart disease.

## Methodology

**Figure 1:**
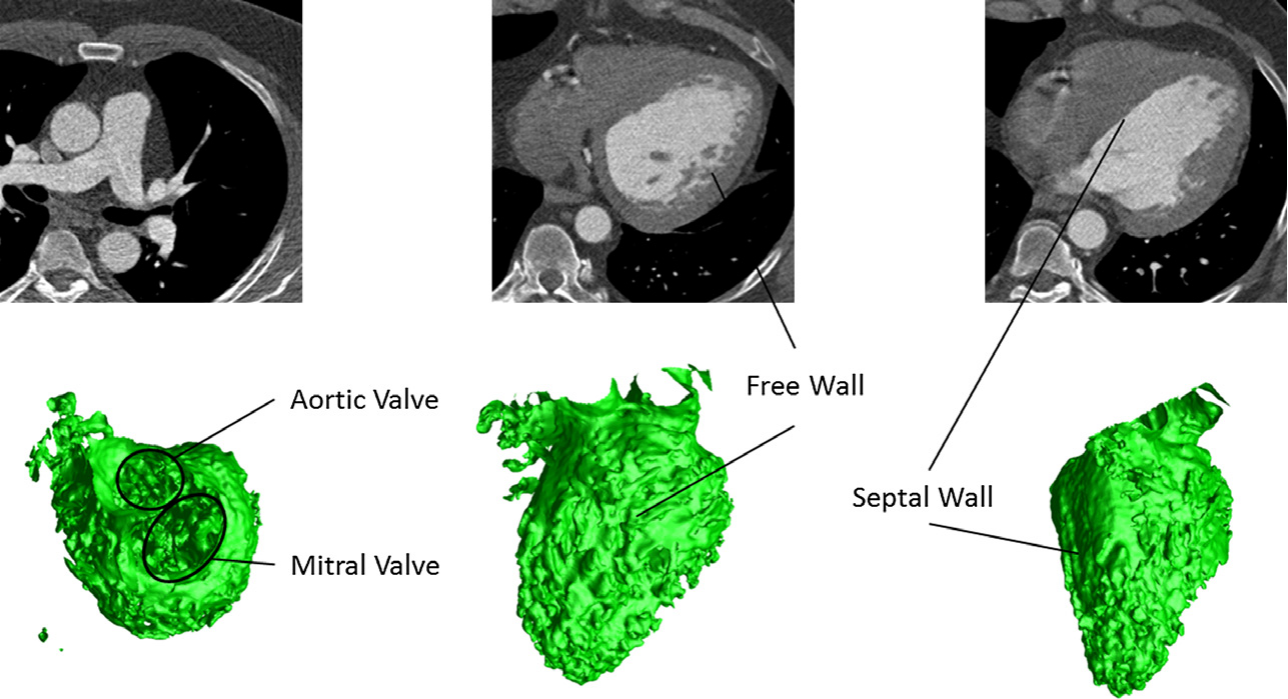
Left ventricular 3D geometry (bottom images) was created from a series of axial CT scan slices (top images) of the heart. Image thresholding and cropping tools were applied to capture only the blood volume within the left ventricle, from which the surface was generated to represent the blood-endocardium interface.

We begin with the notion of the ventricular-blood interface being modeled as a scale-dependent multi-fractal object whose fractal dimension, *D*(λ) is a function of scale size, λ. This interface consists of the outline of VTCs and the papillary muscles. A full 3D model of this surface (see Fig. 1) was acquired at ten equally spaced phases throughout the cardiac cycle using high-resolution cardiac CT imaging at a spatial resolution of 0.77 mm/pixel in a healthy individual. Imaging on a Siemens Definition scanner (Siemens AG, Erlangen, Germany) was phase locked to the patient's ECG signal so that slices were taken at approximately equal time steps of 66 msec, corresponding to 0.66 sec per cardiac cycle. The subject used in this study was a 51-year old male patient with no abnormality in ventricular pumping characteristics. This study was performed with relevant Institutional Review Board approvals. Discretized 3D models of the interface were created for each cardiac phase by segmenting out the blood–endocardium interface using Mimics software (Materialise Inc., Plymouth, MI). While it would be impossible to capture the exact ventricular anatomy at all scales due to CT resolution and image segmentation, these factors only affect very small scale features (<0.5 mm). In this study, the smallest features we are interested in resolving are VTC (∼3–6 mm). Figure 1 shows both the raw CT images and the 3D surfaces visualizing the complexity of the ventricular-blood interface. The meshed models were then imported into MATLAB (MathWorks, Natick, MA) and analyzed using an in-house code to implement the box-counting algorithm (Mandelbrot 1982), which estimates the multi-fractal dimension 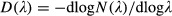, where *N*(λ) is the number of boxes of size *D*(λ), needed to fully cover the interface. *D*(λ) was determined for a range of scales, from ∼0% to 50% of the total/longest length scale of the ventricle. The *D*(λ) profiles obtained as a function of cardiac phase represent the temporal variations in the multi-fractal properties of the VTC interface structure.

**Figure 2:**
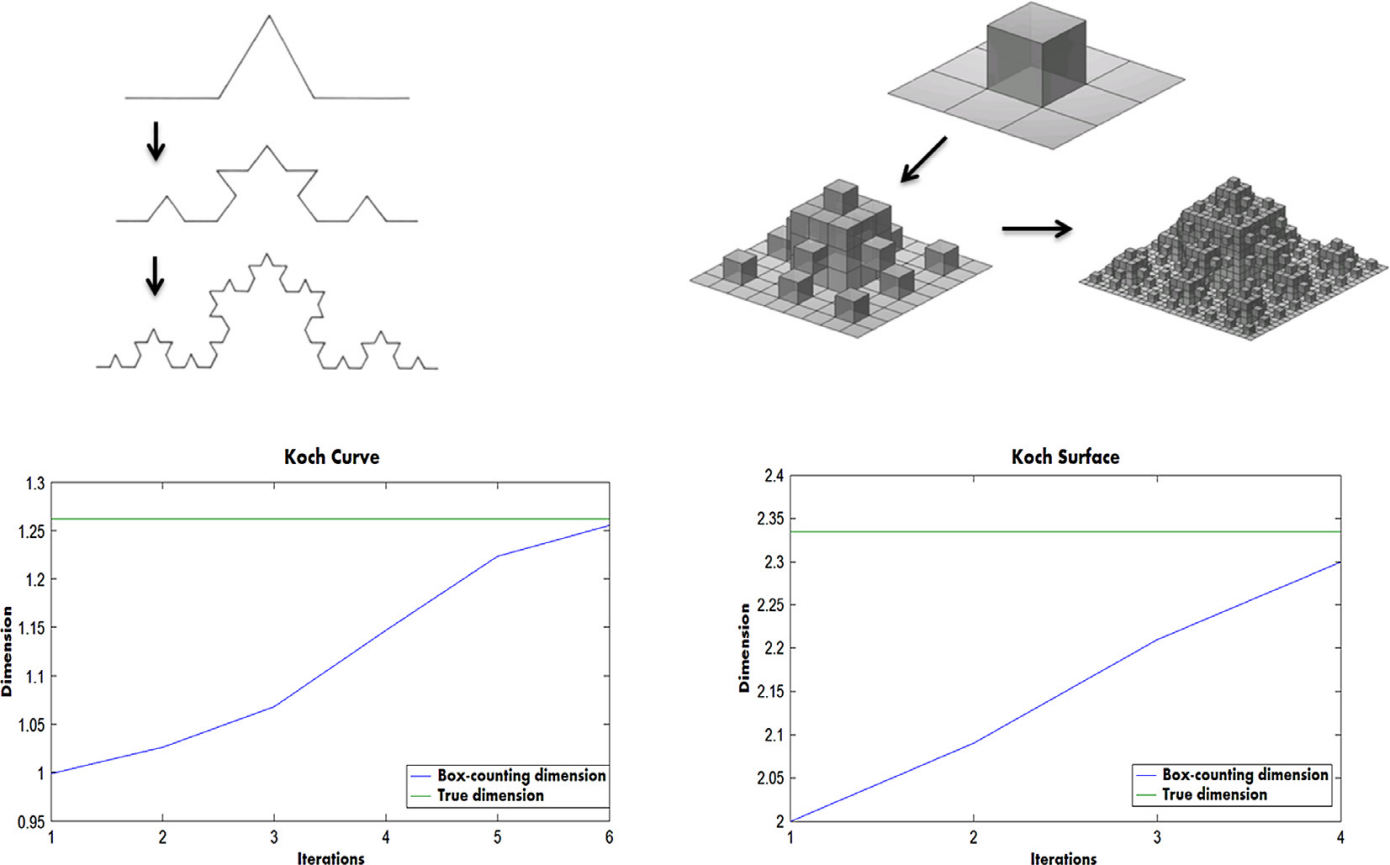
Box-counting validation using two known fractal objects. Objects increase in complexity with each iteration, eventually reaching a theoretical limit. This is accurately reflected by our box-counting algorithm.

A validation study was carried out to verify the accuracy of our in-house code. Fractals with known dimension values were created, and the box-counting dimension was computed and compared to the known dimension. Because these theoretical shapes are generated through an iterative procedure, the box-counting dimension was computed at multiple iterations. Results are presented in Figure 2 and show that the box-counting dimension approaches the true dimension as the number of iterations increases. Theoretically the shapes are created using an infinite number of iterations, but only a few are needed in order to reach an adequate level of complexity for our algorithm to yield a answer correct to within a few percents.

## Results

### General characteristics of VTC dynamics *D*(λ)

**Figure 3:**
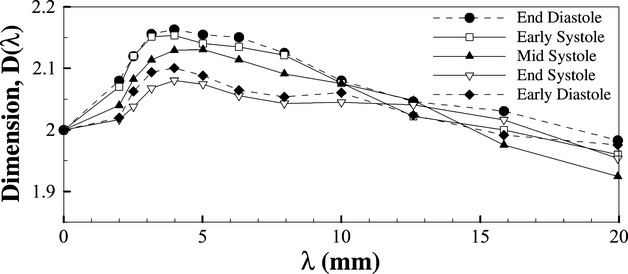
Fractal dimension, *D*, is plotted as a function of scale, λ, at different time points during the cardiac cycle. *D*(λ) is highest around λ = 3–5 mm, corresponding to trabecular length scale. At this length scale, dimension is highest at the start of systole and lowest and the end of systole.

Figure 3 shows the *D*(λ) profiles of the ventricle during five key phases of the cardiac cycle. As seen in the figure, the fractal dimension of the VTC surface is indeed scale dependent. The concave down profiles indicate that the complexity of the interface (i.e., convolutions) is highest at an intermediate scale corresponding to where *D*(λ) profile achieves a local maximum. From the figure *D*(λ) is maximum at relatively low scales where λ ∼3–6 mm. This scale range is consistent with the anatomical size of VTCs structures (Burch et al. 1952). Scale lengths below this range show that the fractal dimension approaches the value of 2.0 consistent with the smooth endocardial surface (relative to the resolution of the image). At the large scales λ > 20 mm, the calculation of fractal dimension using the box-counting algorithm is noted to be unreliable due to the relatively large box sizes compared to the size of the ventricle (data not shown). Between λ ∼15 and 20 mm the fractal dimension is below the value of 2.0 indicating that the surface representation at such large scales begin to have significant gaps. Examining the temporal variation in *D*(λ) illustrate a general temporal trend. During ventricular systole, *D*(λ) consistently decreases with respect to time with the reversal of this trend in ventricular diastole.

### Scale-dependent characteristics of VTC dynamics

**Figure 4:**
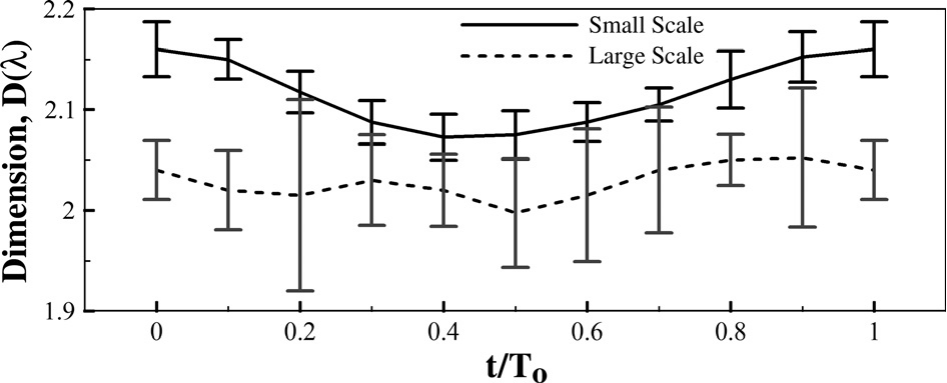
Fractal dimension, *D*, is plotted as a function of normalized time, t/T0 for two different length scales. A significant change in *D* is shown at small scale while no significant difference occurs for large scale features.

To better understand the time-dependent complexity in the shape of VTC structures over the cardiac cycle, we further examine these changes by segregating the temporal response of *D*(λ) averaged over a range of scales. This was achieved by averaging or “binning” the *D*(λ) profiles of Figure 3 into two categories: (1) the small scales, and (2) the large scales, where small scales are defined as λ < 10 mm and the large scales are defined as λ > 10 mm. The temporal response of the average and standard deviation of *D*(λ) defined over these scale ranges are shown in Figure 4. As can be seen in this figure, the dimension of the VTC interface at the small scale significantly varies over the cardiac cycle while that of the large scale does not. Note the significant decrease in dimension (*P* < 0.003) during systolic ejection, which occurs within the initial 40% of the cardiac cycle followed by the more gradual increase in dimension during diastole. The temporal variation in dimension at the large scale is not statistically significant.

**Figure 5:**
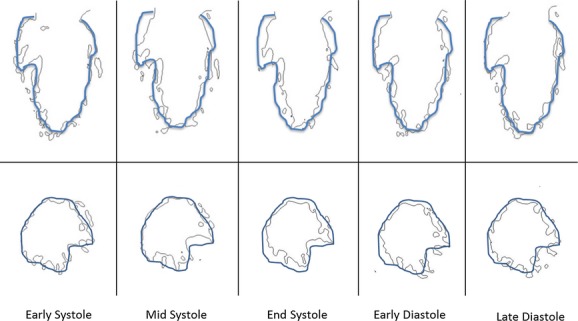
2D slices were extracted from the overall 3D model along the long axis (top) and short axis (bottom) of the ventricle at different times throughout the cardiac cycle. The same thick outline, corresponding to the general shape at the start of systole, is superimposed over each image as a reference for different time points

To relate the above findings through *D*(λ) analysis to observable changes in the geometry of the interface, we present 2D slices along the long and short axes (Fig. 5) for each of the ten cardiac phases. Only five phases (equally spaced in time) are shown in the interest of clarity. In these series of images, the interface can be seen changing from that with an initial rough texture to that with a relatively smoother texture during systole followed by a return to the rough texture in diastole. These instantaneous sections through the complex interface provide visual confirmation of how small-scale features and the overall ventricular wall motion work in synchronized conjunction during systole and diastole. It can be seen that small cavities defined by the convoluted surface of VTCs expel blood during systole. This trend is most evident from early to midsystole, at which point, the walls of the ventricle begin to translate inward. Both of these trends (interface flattening and wall motion) are approximately mirrored during diastole. However, the reappearance of the trabecular structures appears to occur much later toward end diastole.

**Figure 6:**
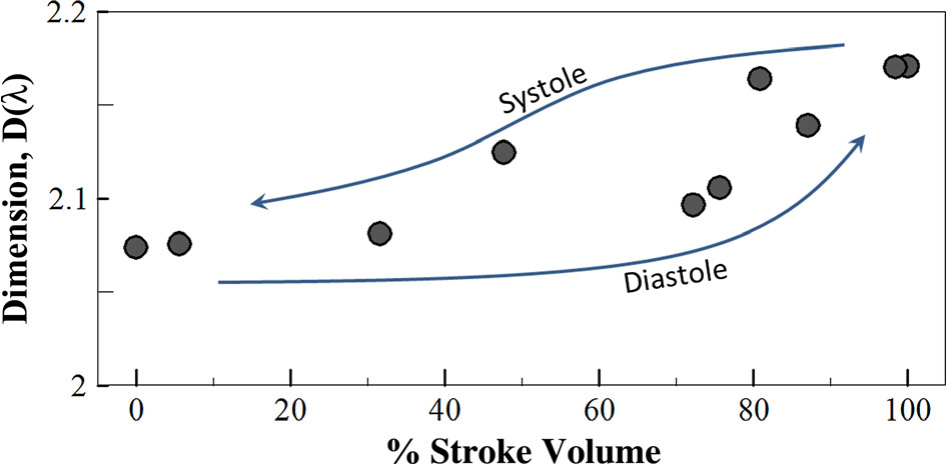
Fractal dimension, *D*, values at ten time points across one cardiac cycle are plotted against stroke volume. *D* is highest when the ventricle is full and lowest at minimum stroke volume, however the path between these two points is different for systole than diastole.

The cyclic changes in the fractal dimension during systole and diastole can be quantitatively related to the overall ventricular contraction by graphing the dimension as a function of ventricular volume. Using the box-counting algorithm, the volume of the ventricle was computed at each cardiac phase. The volume was only calculated for the region within the ventricular cavity by artificially “walling-off” the mitral and aortic annular openings shown in Figure 1. Figure 6 shows the variation in the fractal dimension of the small-scale features only as a function of normalized ventricular stroke volume (D-V loop). It can be seen that the decrease in fractal dimension during systole is not exactly mirrored during diastole based on the apparent hysteresis seen in the cycling of dimension. As seen in the figure, the systolic decrease in fractal dimension appears more gradual with the most rapid reduction occurring midway during the stroke. In contrast, most of the convolutions (i.e., roughness) return during diastole only in the last 30% of the volume stroke.

## Discussion

To our knowledge, the above analysis constitutes the application of fractal geometry to quantify VTC function for the first time. From the CT images we have shown that the small-scale features corresponding to VTCs undergo continuous and quantifiable geometric changes during the cardiac cycle. In this section, we discuss if the results above indicate any new mechanistic role of VTCs other than what is already known (Burch et al. 1952).

The first aspect that will be explored is the effect of scale on fractal dimension. As the VTCs are treated as multi-fractal objects, they may be characterized with fractal dimension as a function of scale. Use of the box-counting algorithm at a certain scale only gives results that describe features around the size of that scale. For example, the ventricle is approximately 120 mL in volume at the end of diastole. In this context, a small-scale feature might be represented by a pocket or cavity between VTCs that encompasses about 5 mL of space, whereas a large-scale feature would be something more descriptive of the overall geometry such as the curvature at the base or apex. The same would hold true for projections, rather than cavities, of similar size.

Notice that the result in the D-V loop shown in Figure 6 where the fractal dimensions of VTCs show “hysteresis” implies that the changes in geometric properties are not just kinematic. It is inferable that the contractions of VTCs are actively modifying the geometric properties throughout the cardiac cycle. Thus, VTCs play a dynamic role as opposed to the more passive role suggested in Ref. (Burch et al. 1952).

**Figure 7:**
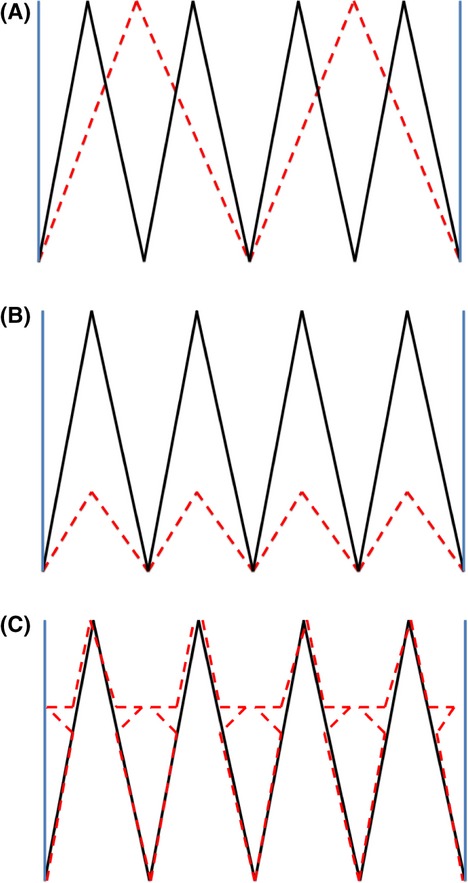
Illustration of three general ways in which an object can change its fractal dimension. Example shows a triangle sine wave changing its (A) frequency, (B) amplitude, and (C) shape. Both (B) and (C) lead to a volume change whereas (A) does not.

Here we attempt to offer a physical explanation behind this observation. The physical argument is that when the VTC interface changes its dimension, it must actively displace blood, and therefore perform a portion of the pressure-volume work/energy transferred to the blood. A physical heuristic argument supporting this notion can be made by examining the definition of fractal dimension as described by the box-counting algorithm (Liebovitch and Toth 1989). As its name suggests, this algorithm involves dividing the 3D-reconstructed space containing the VTC interface into a grid of boxes and then counting the number of boxes that contain at least a part of the VTC surface. Repeating this process for a number of different box sizes and utilizing the relationship 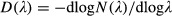, yields the fractal dimension of the surface. As the scale size λ is an independent variable, a change in *D*(λ) means there must be a change in the number of boxes (i.e., *N* ∼ λ^*D*(λ)^). For this to occur, a fractal surface must alter its geometry to take up either more or less space. Because a fractal surface is made up of a number of features that determine its space-filling capacity, it must alter these features to change its box count, and therefore its dimension. A surface can achieve this in three ways: by changing (1) feature frequency, (2) feature size, or (3) feature complexity. By features, we refer to projections or cavities that define the VTCs relative to the overall shape of the ventricular-blood interface. Illustrations of these three ways are depicted in Figure 7. A change in feature frequency as shown in Figure 7A will not yield a change in volume of a region bounded by a fractal surface. To provide a heuristic argument in support of this, let us visualize a fractal interface embedded in 2D appearing as a triangle wave with amplitude *h*, frequency *s*, and spanning an overall length of *L* as shown in Figure 7A. Then the volume (per unit length out of the paper) occupied under each wave is 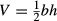, where 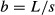. Therefore, total volume, 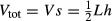, which is frequency independent. However, Figure 7B and C illustrate ways a fractal interface can change its dimension and cause a volume displacement. Because *V*_tot_ is dependent on the amplitude, *h*, of a feature, it shows that variations in feature size indeed cause a volume displacement. Figure 7C is self-explanatory as it shows that a change in feature shape or complexity leads to volume change as this can be represented either by addition or deletion of features.

The above fundamental physical arguments support the notion that VTCs by way of contraction must participate in the pressure–volume work done as they clearly change their size throughout the cardiac cycle. These changes are more clearly shown in Video S1. These small-scale features are essentially small blood-containing cavities along the inner surface of the left ventricle. As the VTCs contract, these pockets flatten out thus expelling the blood that they once contained, as shown by the reduction in fractal dimension in Figure 6. In the absence of VTCs the entire ejection must be accounted for by the overall translation of the ventricular walls. As seen in Figure 5, wall translation occurs simultaneously with the flattening of VTCs. Figure 6 also shows that the VTCs remain flattened through late diastole.

These combined methods of blood displacement, via “squeezing pockets” and wall translation could help overall ventricular function in a manner similar to the way a mechanical transmission system operates. In the case of the transmission, large forces are initially needed to initiate movement, and this is accomplished at the expense of displacement (this is the concept of mechanical leverage). However, with the gain of momentum, the gear ratio can be shifted in order to yield larger displacement without the need for such large forces. The observations presented here are very similar. Systolic ejection of blood appears to be achieved by changing the small-scale geometric features to minimize mechanical effort. The Law of Laplace states that, to develop a certain pressure inside the ventricle, less muscular tension is required for a region of high curvature as opposed to one of low curvature (Burton 1957). As the inner curvature of cavities formed by VTC are large, the relative mechanical forces in the tangential direction need not be great to develop a significant fluid pressure within the cavities thus reducing the effort to initial ejection, which is analogous to starting in a small gear. Once this has occurred, the ventricular walls can then move inward in order to expel the rest of the blood contained in the ventricle, a process corresponding to a higher gear in the mechanical transmission example. During diastole, however, there is no contribution to mechanical effort through this physical mechanism. Therefore, we do not see the VTC projections reappear until late diastole. Thus, VTCs help by providing more leverage at the initiation of ejection.

In summary, we have presented new insight into the physical role of the complex anatomical features called VTCs that are present on the ventricular-blood interface in the human heart. Given the complexity of their geometry, the tools of fractal geometry have been utilized to quantitatively describe the anatomical changes in VTC geometry over the cardiac cycle. We show that the significant changes in multi-fractal dimension at the VTC length scales describe a mechanism to enhance mechanical leverage to initiate ejection during early systole. These results are the first of its kind to describe the complex biomechanical role of VTCs in the physiology of ventricular ejection phase. Future work is obviously needed to better understand the clinical relevance of the above findings with respect to factors such as age, gender, status of heart disease, etc. in large patient databases. The limitation of this study is that it only offers a novel hypothesis regarding the potentially new mechanical role of VTCs in cardiac pumping.
